# Genome-wide SNP identification in multiple morphotypes of allohexaploid tall fescue (*Festuca arundinacea* Schreb)

**DOI:** 10.1186/1471-2164-13-219

**Published:** 2012-06-06

**Authors:** Melanie L Hand, Noel Oi Cogan, John W Forster

**Affiliations:** 1 Department of Primary Industries, Biosciences Research Division, Victorian AgriBiosciences Centre, 1 Park Drive, La Trobe University Research and Development Park, Bundoora, VIC 3083, Australia; 2 Dairy Futures Co-operative Research Centre, Bundoora, Australia; 3 La Trobe University, Bundoora, VIC 3086, Australia

**Keywords:** *Lolium arundinaceum*, Molecular marker, DNA sequencing, Haplotype, Sub-genome

## Abstract

**Background:**

Single nucleotide polymorphisms (SNPs) provide essential tools for the advancement of research in plant genomics, and the development of SNP resources for many species has been accelerated by the capabilities of second-generation sequencing technologies. The current study aimed to develop and use a novel bioinformatic pipeline to generate a comprehensive collection of SNP markers within the agriculturally important pasture grass tall fescue; an outbreeding allopolyploid species displaying three distinct morphotypes: Continental, Mediterranean and rhizomatous.

**Results:**

A bioinformatic pipeline was developed that successfully identified SNPs within genotypes from distinct tall fescue morphotypes, following the sequencing of 414 polymerase chain reaction (PCR) – generated amplicons using 454 GS FLX technology. Equivalent amplicon sets were derived from representative genotypes of each morphotype, including six Continental, five Mediterranean and one rhizomatous. A total of 8,584 and 2,292 SNPs were identified with high confidence within the Continental and Mediterranean morphotypes respectively. The success of the bioinformatic approach was demonstrated through validation (at a rate of 70%) of a subset of 141 SNPs using both SNaPshot™ and GoldenGate™ assay chemistries. Furthermore, the quantitative genotyping capability of the GoldenGate™ assay revealed that approximately 30% of the putative SNPs were accessible to co-dominant scoring, despite the hexaploid genome structure. The sub-genome-specific origin of each SNP validated from Continental tall fescue was predicted using a phylogenetic approach based on comparison with orthologous sequences from predicted progenitor species.

**Conclusions:**

Using the appropriate bioinformatic approach, amplicon resequencing based on 454 GS FLX technology is an effective method for the identification of polymorphic SNPs within the genomes of Continental and Mediterranean tall fescue. The GoldenGate™ assay is capable of high-throughput co-dominant SNP allele detection, and minimises the problems associated with SNP genotyping in a polyploid by effectively reducing the complexity to a diploid system. This SNP collection may now be refined and used in applications such as cultivar identification, genetic linkage map construction, genome-wide association studies and genomic selection in tall fescue. The bioinformatic pipeline described here represents an effective general method for SNP discovery within outbreeding allopolyploid species.

## Background

The increased scale of data generation and reduction in associated costs characteristic of second-generation sequencing technologies has recently permitted large-scale single nucleotide polymorphism (SNP) discovery efforts for crop species that previously had limited genomic resources. SNPs have rapidly become the molecular genetic marker of choice for a range of applications, largely due to their abundance within the genome, co-dominant nature and amenability to high-throughput detection that produces results with low error rates [1-3]. An extensive resource of SNPs distributed throughout the genome will potentially allow the construction of high-density genetic maps, genome wide association studies (GWAS) and genomic selection (GS) to be performed in the targeted species [4,5]. Although large-scale SNP collections were once confined to the most economically valuable crops such as maize and rice [6-9], reports have now been made for species with traditionally fewer genomic resources including alfalfa [10], common bean [11], Amaranth [12], oat [13] and sugarcane [14].

All SNP discovery activities rely on the resequencing of targeted regions from single or multiple genotypes, alignment of the sequence reads and identification of nucleotide variants within and between the genotypes. More specifically, in plant species, this process has generally been accomplished through sequencing of transcriptomes [15,16], PCR amplicons [14], gene spaces enriched by various methods [15] and genomic fractions of reduced complexity obtained using methods such as restriction endonuclease digestion and size fractionation, or enrichment for hypomethylated genomic regions [11,12,15-17]. Regardless of the methodology used, the process of SNP discovery is considerably more complicated for allopolyploid species, as interpretation of sequence data is hindered by the presence of multiple, often divergent, sub-genomes. Discrimination must then be performed between the nucleotide variation representing differences between these sub-genomic copies (homoeologous sequence variants [HSVs]) [18] and the homologous allelic variation arising within or between individuals, that is able to be assayed as a polymorphic molecular genetic marker (SNP) [19]. For plants, this process is most challenging in outbreeding species with multiple heterozygous genomic constitutions, for which nucleotide variation within each resident sub-genome further complicates data interpretation. Subsequent SNP genotyping is also more complicated for polyploid as compared to diploid taxa. Quantitative genotyping methods are required to fully classify nucleotide variant dosage and hence verify the genotype at any given locus or homoeolocus [19].

As an outbreeding allohexaploid (2n = 6x = 42) species, the agronomically important pasture grass tall fescue (*Festuca arundinacea* Schreb. syn. *Lolium arundinaceum* (Schreb.) Darbysh.) provides a clear example of complex plant genome architecture. To date, molecular marker development within tall fescue has been restricted to either anonymous marker systems such as amplified fragment length polymorphisms (AFLPs), laborious sequence-specific systems such as restriction fragment length polymorphisms (RFLPs), or more efficient and informative simple sequence repeat (SSR) assays based on analysis of either expressed sequence tag (EST) or genomic DNA sequences [20-22]. More recently, a high-throughput genotyping system designed for *Lolium* and *Festuca* species was developed in the form of a Diversity Arrays Technology (DArT) array [23]. However, for improvement of tall fescue through GWAS or GS, a collection of SNPs distributed throughout the genome is ultimately desirable.

All molecular genetic marker development performed to date in tall fescue has been focused on the Continental morphotype, which represents only one of three currently recognised eco-geographic races of tall fescue. Continental tall fescue has historically been domesticated into the most commonly cultivated tall fescue varieties, however two other morphotypes (Mediterranean and rhizomatous) have also been recognised. The contemporary species believed to be most closely related to the putative progenitors of Continental tall fescue have been identified as the diploid (2x) taxon meadow fescue (*F. pratensis* Huds. syn. *L. pratense* (Huds.) Darbysh.) and a tetraploid form (4x) of tall fescue (*F. arundinacea* var. *glaucescens* syn. *F. arundinacea* subsp. *fenas*) [24]. Recent advances suggest that Mediterranean tall fescue has evolved independently in Northern Africa following the hybridization of a different set of progenitor species, which have not been identified. Mediterranean tall fescue may hence be better described as a separate taxon from Continental tall fescue [25]. Conversely, current evidence suggests that the rhizomatous and Continental tall fescue morphotypes share the same progenitor species, but have since undergone genomic divergence [25].

This study aimed to assemble a collection of SNPs distributed throughout the tall fescue genome, based on resequencing amplicons that are predicted to be located at regular intervals across each homoeologous group. Specifically, novel bioinformatic approaches were developed to reduce the complexity of sequence contigs and ultimately to identify SNPs within specific sub-genomes. Given the demonstrated differences between genomic constitutions of Continental and Mediterranean tall fescues, the experimental procedures were applied in parallel to representatives of each group, as well as the rhizomatous morphotype.

## Methods

### Plant materials

Amplicons for DNA sequencing were generated from 12 tall fescue genotypes, six of which were from the Continental morphotype, five from Mediterranean, and one from rhizomatous (Table 1). Non-cultivar material was obtained from the United States Department of Agriculture (USDA) tall fescue germplasm collection and the morphotype identity of each accession has been previously determined [26]. An equivalent amplicon set was also generated from a diploid meadow fescue accession (Bf 1199) and a *F. arundinacea* var. *glaucescens* accession (Bn 581), both which were obtained from the Genetic Resources Unit, Institute for Biological, Environmental and Rural Studies (IBERS), Aberystwyth, Wales. Additional genotypes obtained from the USDA tall fescue collection were used for SNP validation (Additional File 1), including representatives of each tall fescue morphotype, and Continental tall fescue genotypes displaying alternate *matK* chloroplast DNA sequence haplotypes, as determined previously [26]. The validation panels were designed to represent a diverse selection of tall fescue, as determined by SSR markers in previous work [26].

**Table 1:** Sequencing output for each tall fescue genotype included

**Morphotype/species**	**Genotype**	**No. sequence reads**	**No. Amplicons sequenced (%)**	**Average reads per amplicon**
Continental tall fescue	Jesup	42730	393 (95)	122
	KY31	40920	394 (95)	113
	Quantum	5490	377 (91)	16
	PI 422743	27361	386 (93)	77
	PI 287820	52711	405 (98)	146
	PI 422714	52580	405 (98)	142
Mediterranean tall fescue	Prosper	25042	397 (96)	72
	Resolute	3231	365 (88)	9
	PI 287819	16632	365 (88)	51
	PI 269850	27005	356 (86)	88
	PI 598932	34231	403 (97)	97
Rhizomatous tall fescue	Torpedo	42076	347 (84)	138
*F. arundinacea* var. *glaucescens*	BN 581	3778	361 (87)	12

### Amplicon design and generation

The amplicons sequenced were selected based upon their likelihood to be evenly distributed throughout the tall fescue genome, as determined by a comparative genome analysis involving wheat (*Triticum aestivum* L.), perennial ryegrass (*Lolium perenne* L.) and *Brachypodium distachyon*. The comparative sequence analysis between both wheat deletion bin-mapped ESTs [27] (Additional File 2) and perennial ryegrass ESTs [28] and the *B. distachyon* 8X genome release [29] was performed using the BLASTN search tool (best hits retained with e-value < 10^-20^) [30]. All primers were subsequently designed based on either wheat or perennial ryegrass EST template sequences using Primer3 software [31], and were intended to amplify regions approximately 500 bp in length.

PCRs were performed in a total volume of 10 μl, and contained 1 x Immolase PCR buffer, 1.5 mM MgCl_2_, 200 μM dNTPs, 0.25 μM each primer, 0.2 units Immolase DNA polymerase (Bioline) and approximately 5 ng of genomic DNA template. Cycling conditions for the perennial ryegrass EST-derived amplicons were as follows: an enzyme activation step of 95°C for 15 min, 30 cycles of 95°C for 1 minute, 55°C for 1 minute, 72°C for 1 minute and a final extension of 72°C for 7 minutes. Amplicons generated using primers designed from wheat EST sequence template used an alternative annealing temperature of 60°C.

Individual PCR products were purified with the addition of Agencourt AMPure® (Beckman Coulter) solution equivalent to 1.7 x the prior reaction volume. The magnetic beads were subsequently washed, and DNA was eluted according to the manufacturer’s protocol. A 30-fold dilution of the purified product was quantified using PicoGreen® (Life Technologies) following the manufacturer’s instructions. Amplicons were subsequently pooled to obtain an approximate equimolar concentration of each amplicon, based on the relative DNA concentration as estimated by PicoGreen® (Life Technologies) and the amplicon length. A separate pool of amplicons was generated for each genotype processed, resulting in a total of 13 pools.

### Sequencing library preparation

Pooled amplicons were concentrated using the MinElute PCR Purification Kit (Qiagen) and 5 μg of each pool was sheared for 65 seconds using the Covaris S2 ultra-sonicator with the duty cycle set to 5%, an intensity of 3, and 200 cycles per burst. Sheared samples were purified using the MinElute PCR Purification Kit (Qiagen) and 5 μg of each sample was loaded onto a 1% (w/v) agarose-TAE gel and electrophoresed at 100 volts for 90 minutes to allow fragment separation. Fragments 400 to 700 bp in size were excised from the gel and purified using the MinElute Gel Extraction Kit (Qiagen), followed by further purification with the Agencourt AMPure® (Beckman Coulter) reagent. Unique 8 bp sequence tags were ligated to the fragments of each amplicon pool to allow the genotype origin of each sequence read to be identified following pooling of all amplicons. This process of barcoding was performed as described by a previous study [32]. When tagged samples were pooled, 150 ng of each hexaploid tall fescue genotype was pooled together along with 100 ng of the tetraploid *F. arundinacea* var. *glaucescens* genotype.

Following ligation of double stranded GS FLX Titanium A and B adapters (Roche), the library was PCR amplified in a total volume of 30 μl containing 1 x Phusion buffer, 200 μM dNTPs, 0.2 μM each A and B primer, 0.6 units Phusion High-fidelity DNA Polymerase (Finnzymes, Thermo Fisher Scientific) and 20 ng input DNA. Cycling conditions included a hot start of 98°C for 30 seconds followed by 15 cycles of 98°C for 10 seconds, 58°C for 20 seconds and 72°C for 60 seconds. Following purification using the MinElute PCR Purification Kit, the amplified library was assessed by loading a 1 μl aliquot on a Agilent Bioanalyzer 1000 DNA chip according to the manufacturer’s instructions.

## 454 GS FLX sequencing

The amplicon library was clonally amplified by emulsion PCR as described in the emulsion PCR (emPCR) Method Manual Lib-L SV, GS FLX Titanium Series (October 2009, Rev. Jan 2010 [Roche]). The emPCR titration method, as described in the same manual, was used to determine the optimal volume of amplicon library to use for emPCR amplification, such that 10-12% of the capture beads become enriched. A total of 1.58 million enriched capture beads were deposited on two regions of a four-region gasket and sequenced using the GS FLX Titanium instrument (Roche). Sequence reads were subjected to run-time data processing according to default parameters defined within the GS FLX Titanium processor. Only sequence reads that passed this quality filter were accessible for analysis.

### Sanger sequencing

The set of amplicons obtained from diploid meadow fescue accession Bf1199 were purified and directly sequenced using dideoxynucleotide chain termination (Sanger) chemistry as described previously [25].

### Sequence assembly and SNP identification

Sequence reads were initially sorted by barcode, and then the barcode and adaptor sequences were trimmed from each read using NextGENE software (Softgenetics). Amplicon coverage was estimated for each genotype using a BLASTN search [30] to query the sequence reads with the perennial ryegrass and wheat EST template sequences using an e-value threshold of 0.001. Sequence reads from tall fescue cultivars Jesup, Prosper and Torpedo were assembled *de novo* using the MIRA assembly software [33]. Assembly parameters were stringently designed to assemble contigs representing sub-genomes within the allohexaploid genome. Required percentage was set to 97%, with a minimum of 100 overlapping base pairs required. A minimum of 5 reads were required per contig, and singletons were saved. Reads from the tetraploid *F. arundinacea* var. *glaucescens* genotype were also assembled *de novo* using the same parameters with the exception of minimum reads per contig, which was reduced to 2 in consideration of the lower number of reads generated for this species. The amplicon origin of each contig was determined by mapping of their consensus sequence to the meadow fescue genome-derived amplicon sequences using MIRA software at a lower stringency of 80% percentage required, in order to co-assemble each putative sub-genome consensus. Those contigs with no match to a meadow fescue template sequence were further mapped to the perennial ryegrass and wheat ESTs originally used as template for primer design.

NextGENE software (Softgenetics) was used to align all other sequence reads to the reference contig assembly of the appropriate morphotype. Each genotype was assembled separately with a matching requirement of greater than 50 bp overlap at 80% identity or more specified. All nucleotide variants within each contig were recorded in the generated SNP report. Assembly of putative sub-genome consensus sequences to identify putative HSVs and diagnostic SNPs was performed using the mapping component of MIRA software [33] with the percentage required set to 80% and a minimum of 10 overlapping base pairs required. Nucleotide positions identified as containing HSVs were subsequently removed from the SNP reports prior to any calculations of SNP numbers. Nucleotide variants were identified as putative SNPs when they were present in greater than one genotype (but not all genotypes) and showed no co-location with putative HSVs. No indels were considered as putative SNPs.

### SNP validation using SNaPshot™ chemistry

Putative SNPs were chosen for validation when they were positioned within the middle of a contig (in order to avoid variants arising from misalignment at the termini of each contig). Interrogation primers were designed adjacent to each variant base to enable implementation of the SNaPshot™ assay (Life Technologies). Loci containing the SNP of interest were initially amplified using PCR as described previously and purified with the addition of Exonuclease I and Shrimp Alkaline Phosphatase (SAP). The SNaPshot™ reaction was performed in a total volume of 5 μl which contained 1 μl of SNaPshot™ Reaction Mix, 0.1 μM interrogation primer, and 1 μl of purified PCR product as template, and was subjected to cycling conditions as described in the associated manual. Each fluorochrome-labelled extension product was purified with the addition of SAP, and subsequently diluted 8-fold with H_2_O. A total of 2 μl of this dilution was combined with 8.95 μl of Hi-Di™ formamide (Life Technologies) and 0.05 μl of GeneScan™ 120 LIZ® Size Standard (Life Technologies) and genotyping was performed using the ABI3730xl capillary electrophoresis platform (Life Technologies). Analysis was completed using GeneMapper Software version 3.7 (Life Technologies).

### SNP validation using GoldenGate™ chemistry

The GoldenGate™ assay was performed according to the manufacturer’s protocol and used the Illumina universal bead-chip which was scanned by the iScan system, following the manufacturer’s directions. Allele calling was performed using GenomeStudio v2011.1 (Illumina). This software calculates the intensity of the Cy3 and Cy5 fluorescence, where the two fluorochromes represent the two alleles, and uses the Cy3/Cy5 ratio to determine the genotype of each individual at a particular locus. Those SNPs identified as polymorphic were categorized based on their observed clustering behaviour. As a measure of SNP performance, the generated allele calls were compared to those obtained using SNaPshot™ chemistry. This comparison considered differences created by the co-dominant genotyping capacity of the GoldenGate™ chemistry and did not consider missing data.

### Diversity analysis

SNP scores from the SNaPshot™ validation assay were used to estimate the genetic similarity of the tall fescue genotypes within the validation panel. The alleles were converted to a binary scoring system for each SNP locus and the Dice [34] coefficient was used to generate a similarity matrix in the program DARwin 5.0.158 [35]. A phenogram was constructed from the genetic distances by the unweighted group method of arithmetic averages (UPGMA). The genetic relationships were compared to those generated using 302 alleles scored from 28 SSR markers. SSR allele data was available for the genotypes within the SNP validation panel following an earlier study [26].

### Sub-genome attribution

Sequence reads derived from Continental tall fescue cv. Jesup, meadow fescue and *F. arundinacea* var. *glaucescens* were aligned using Sequencher 4.7 (Gene Codes) for each amplicon, and contigs were trimmed to make each read equal length. Neighbour-joining trees were constructed using MEGA version 4 [36] with the maximum composite likelihood nucleotide substitution model.

## Results

### Design of amplicons from genome-wide distributed genomic loci

Genomic regions predicted to be conserved between members of the Pooideae subfamily were initially determined through comparison of the chromosomal locations of wheat deletion bin-mapped ESTs with their putative orthologues in the genome of the model sequenced model grass, *B. distachyon*. A total of 19 blocks of conserved synteny between the wheat and *B*. *distachyon* genomes were recorded (Additional Files 3 and 4), and it was inferred that these genomic regions are also likely to be conserved between perennial ryegrass and *B*. *distachyon*. Perennial ryegrass ESTs were subsequently used to query the *B*. *distachyon* genome draft in order to identify loci that are likely to be physically located within the previously defined macrosyntenic blocks. A total of 668 genomic loci were used for primer design to generate amplicons, of which 321 successfully amplified a single product within hexaploid tall fescue. An additional 93 primer pairs designed directly from wheat EST template proved to also successfully amplify single products from tall fescue, resulting in a total of 414 amplicons selected for 454 GS FLX sequencing. The predicted distribution of these 414 amplicons is given in Additional File 5.

### Sequencing and reference contig assembly

454 GS FLX sequencing from two regions of a four-region gasket produced 379,060 sequence reads that passed the run-time quality filter. Sequencing output was variable across sequenced genotypes, with a maximum of 52,711 (PI 287820) and minimum of 3,231 (Resolute) sequence reads achieved per genotype (Table 1). This variability is likely to be associated with differences in efficiency of each individual barcode ligation reaction. A comprehensive coverage of the amplicon set was achieved for each genotype (84-97% coverage) with the sequencing depth for each amplicon ranging from 9 to 146 sequence reads per genotype, and reflecting the variability of the sequencing output (Table 1). Sequence reads from all genotypes were submitted to the short read archive in GenBank (http://www.ncbi.nlm.nih.gov) and were assigned the accession number SRA052217. Sanger sequencing of the amplicon set from meadow fescue produced single reads with no overlapping double peaks for 253 (61%) of the amplicons. The remainder either failed to be amplified from the meadow fescue genotype, or, when sequenced, produced mixed reads indicative of multiple product amplification.

Reads derived from sequencing templates of cultivars Jesup (Continental morphotype) and Prosper (Mediterranean morphotype) were assembled individually *de novo* using parameters stringently designed to assemble contigs representing sub-genomes within the hexaploid. This *de novo* assembly yielded 1,270 and 712 contigs, respectively (Table 2), which represented an average of 2.54 and 2.03 contigs per amplicon. However, up to 9 contigs were assembled for some amplicons. The consensuses of these contigs are subsequently designated as putative sub-genome consensus sequences. For Jesup and Prosper, these putative sub-genome consensus sequences were assembled both within each cultivar and between the two cultivars to identify putative HSVs internal to each morphotype, as well as potential nucleotide variants that are diagnostic for (fixed within but differing between) the Continental and Mediterranean morphotypes. Following this assembly process, an HSV was predicted to occur every 109 bp within amplicons from Jesup, and every 122 bp within amplicons from Prosper (Table 2).

**Table 2:** **Characteristics of the continental tall fescue (Jesup) and mediterranean tall fescue (Prosper)*de novo*reference assemblies**

	**Jesup**	**Prosper**
Number of contigs	1270	712
Average contig length (bp)	558	520
Average reads per contig	14	11
Average contigs per amplicon	2.54	2.02
Average HSVs per amplicon	20	15
HSV frequency	1 HSV every 109 bp	1 HSV every 122 bp.

### Sequence alignment and SNP discovery

Using the Jesup and Prosper putative sub-genome consensus sequences as references for the Continental and Mediterranean tall fescue genotypes respectively, sequence reads from the remaining genotypes were aligned, with the aim of detecting SNPs within sub-genomes of each individual genotype. The number of contigs within the Continental reference sequence that were covered by the remaining genotypes ranged from 70% (Quantum) to 97% (PI 287820), while the degree of coverage of the Mediterranean reference varied from 60% (Resolute) to 97% (PI 598932). A file listing only unique SNP positions was compiled based upon the generated SNP reports from each genotype. SNPs within each contig that co-located with the previously identified putative HSVs were removed from the list.

By comparing the generated SNP reports for each of the six Continental tall fescue genotypes, a total of 18,890 mutations were detected (Table 3). However, when indels were eliminated and SNPs present in only more than one genotype were retained, 8,584 high-confidence SNPs remained, equating to 15 per amplicon sequenced. Fewer SNPs were detected for the Mediterranean morphotype after comparison of the reports for the five studied genotypes. A total of 7,631 mutations were identified, but only 2,292 high-confidence SNPs remained following indel removal and qualification based on presence in multiple genotypes (Table 3). An average of almost 8 SNPs per amplicon sequenced was predicted for the Mediterranean morphotype.

**Table 3:** Summary of polymorphic SNPs predicted within each tall fescue morphotype and diagnostic SNPs identified between morphotypes

	**Continental**	**Mediterranean**	**Diagnostic C/M**^**1**^	**Diagnostic C/R**^**2**^
Number of SNPs	18890	7361	3004	1481
Number of indels	12884	4877	431	2108
Number of SNPs(in >1 genotype)	8584	2292	-	-
Average SNPs per sub-genome contig	8.36	6.06		
Average SNPs per amplicon	14.68	7.73	7.72	5.84

^1^ SNPs diagnostic between Continental and Mediterranean morphotypes.

^2^ SNPs diagnostic between Continental and Rhizomatous morphotypes.

Nucleotide variants capable of diagnostic differentiation between the Continental and Mediterranean tall fescue morphotypes were also predicted using the Jesup and Prosper-derived putative sub-genome consensus sequences (Table 3). Those variants detected between the two reference sets that further appeared to be monomorphic within the sequenced genotypes were tentatively classified as diagnostic in nature. A total of 3,004 of such sequence variants were identified between the Continental and Mediterranean morphotypes, while 1,481 were potentially able to differentiate Continental and rhizomatous tall fescue germplasm.

### SNP validation using SNaPshot™ chemistry

A total of 96 putative SNPs (from 81 different amplicons) chosen for validation using SNaPshot™ chemistry were predicted to be polymorphic within either the Continental (59) or Mediterranean (19) morphotypes. The remainder were predicted to be diagnostic nucleotide variants capable of discrimination between the Continental and Mediterranean morphotypes (9) or the Continental and rhizomatous morphotypes (9).

SNP validation using SNaPshot chemistry was designed to identify HSVs that had been mis-classified as putative SNPs, due to inadvertent passage through the computational filters, by empirical screening over a large collection of diverse germplasm. Of the 96 SNPs selected for validation across 48 diverse tall fescue genotypes, 89 were successfully assayed and 70 (73%) were polymorphic across the 48 genotypes (Table 4). Of those SNPs selected from the Continental tall fescue genotypes, 81% were polymorphic, while 68% of the Mediterranean tall fescue-derived class were polymorphic. A single nucleotide variant selected from this class also proved capable of differentiating the Continental and Mediterranean genotypes included in the validation panel. The validation rate of the 18 putative diagnostic variants was lower than that of the other two classes, with only 3 (17%) proving diagnostic within the validation panel. The remaining 15 nucleotide variants in this class were either revealed as polymorphic within this diverse panel (50%), monomorphic between morphotypes (17%) or were unable to be assayed (17%). Details of the SNaPshot™ assays and the resulting allele calls are supplied in Additional Files 6 and 7. In order to interpret the success rates of the validated SNP assays, the read depth of each original contig used for SNP prediction was examined. However, no correlation between contig depth and level of SNP validation was observed.

**Table 4:** Results of SNP validation using SNaPshot™ and GoldenGate™ chemistries including a comparison of the genotype calls

**SNP class and sub-class (GoldenGate clustering)**	**Number of SNPs**			**% genotypes with concordant allele calls**
	**SNaPshot**	**GoldenGate**	**Compared between genotyping systems**^**1**^	
Polymorphic	70	62	38	80.45
3 clusters (1 sub-genome amplified)	N/A	17	12	87.01
3 clusters (3 sub-genomes amplified)	N/A	11	5	86.00
2 clusters	N/A	20	13	80.15
5 clusters	N/A	2	1	48.65
Dispersed	N/A	12	7	71.71
Diagnostic	4	3	1	97.67
Monomorphic	15	16	7	39.11
Failed	7	14	5	N/A
Total	96	96	51	76.43

^1^ SNPs are classed according to the GoldenGate™ results.

### SNP validation using GoldenGate™ chemistry

A collection of 96 SNPs (from 81 different amplicons) was used to test the efficiency of SNP genotyping in tall fescue using Illumina GoldenGate™ chemistry. The SNPs selected were predicted to be polymorphic within either the Continental (58) or Mediterranean (22) morphotypes. The remainder were again predicted to be diagnostic between the Continental and Mediterranean morphotypes (6) or the Continental and rhizomatous morphotypes (10). Of these 96 SNPs, 45 were previously included in the SNaPshot™ validation experiment.

SNP genotyping using the Illumina GoldenGate™ assay was performed with the aim of further validation, while also determining the capacity for quantitative genotyping in hexaploid tall fescue. Of the 96 loci assayed, polymorphic clustering was observed among the included genotypes for 62 (Table 4), which were therefore defined as validated and of high quality. No predicted diagnostic variants were validated using GoldenGate™ chemistry, nonetheless, 3 loci predicted as polymorphic SNPs within either the Continental or Mediterranean morphotypes were ultimately identified as diagnostic for morphotype in the validation panel. Inclusion of genotypes from all three morphotypes also allowed a cross-comparative analysis of the SNP performance in each morphotype. A greater proportion of putative SNPs were validated when assayed from the morphotype from which they originated, however, some level (45 – 48%) of cross-transfer between the Continental and Mediterranean tall fescue morphotypes was observed. Details of the GoldenGate™ assay and the resulting allele calls are supplied in Additional Files 6 and 8.

**Figure 1:**
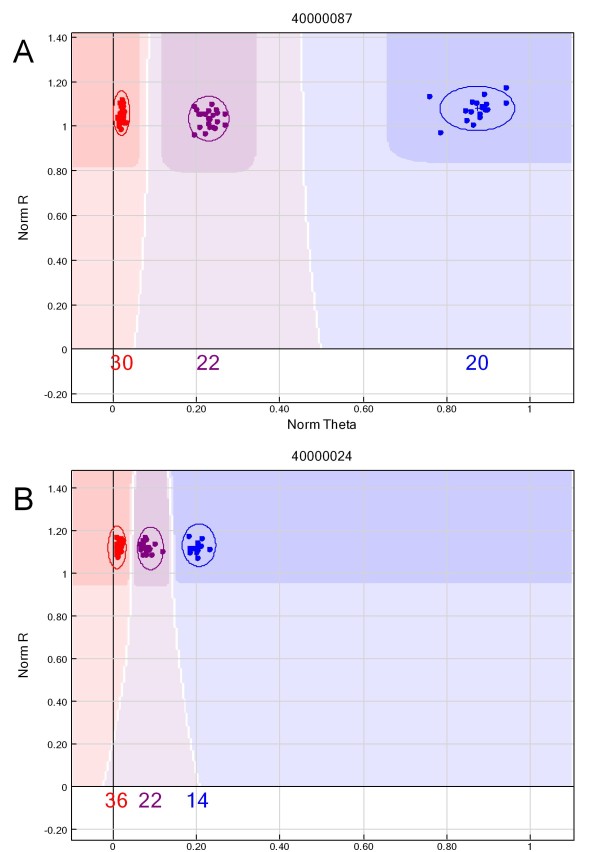
**Co-dominant SNP genotyping.** An example of genotype plots generated by GenomeStudio following the GoldenGate™ assay, using 64 Continental and 8 rhizomatous tall fescue individuals. For 17 SNPs, the loci may possibly have been amplified only from a single sub-genome and generated Cy3/Cy5 ratios close to 1:0 and 0:1, as may be expected for a diploid species (**A**). Co-dominant genotyping was still achievable for others (11), despite amplification from multiple sub-genomes altering the Cy3/Cy5 ratio (**B**).

Despite the presence of multiple sub-genomes, the quantitative genotyping capacity of the GoldenGate assay permitted a reasonable proportion (30%) of tested SNPs to be scored in a co-dominant fashion. For 17 of these, the target loci appear to be amplified from only one sub-genome, as the Cy3/Cy5 ratios for the homozygotes were approximately 1:0 and 0:1, as is characteristic of diploid species. For 11 loci, all sub-genomes were probably amplified, as the Cy3/Cy5 ratios were clearly skewed. However, the quantitative capacity of this assay still permitted the three genotype classes to be clearly distinguished (Figure 1). The remaining polymorphic SNPs either formed two clusters (typically consisting of one homozygous cluster and one larger, more diffuse heterozygous cluster), five clusters, or more than three, less distinct clusters, where samples were observed to have a broad spectrum of Cy3/Cy5 ratios (classed as dispersed).

A comparison of the allele calls generated using the SNaPshot™ and GoldenGate™ chemistries could be performed for 51 SNPs across 44 genotypes (Table 4). In total, an average of 76% of genotypes yielded identical allele calls from both genotyping chemistries, although variability was observed, relating to the performance of the SNP in the GoldenGate™ assay. The highest degree of concordance was achieved when the SNP was either diagnostic (97.67%), or formed three distinct clusters (86.00 – 87.01%). Conversely, the largest amount of discordance was observed for SNPs identified as monomorphic within the GoldenGate™ assay (39.11%).

### Assessment of SNP-based genetic diversity

**Figure 2:**
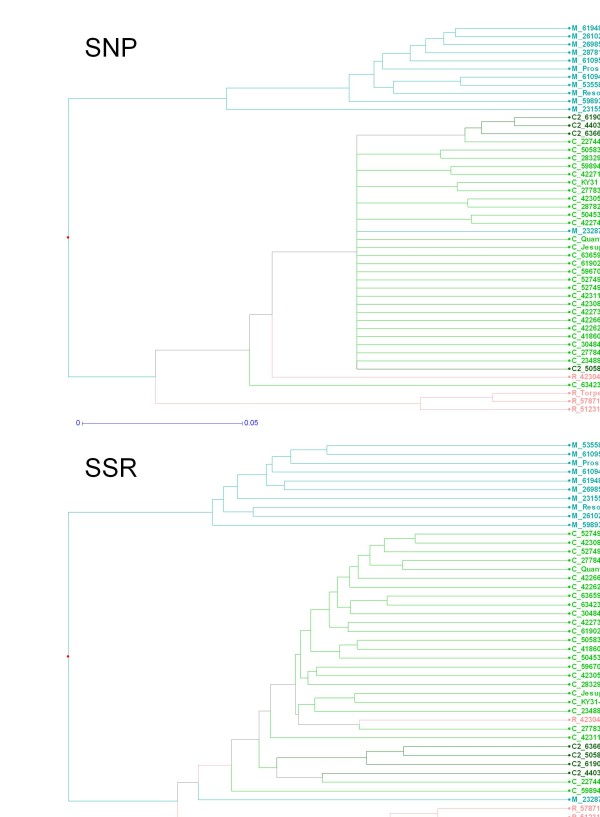
**Diversity analysis.** Unweighted group method of arithmetic averages (UPGMA) phenogram constructed using allele scores of 64 SNP (top panel) or 28 SSR (bottom panel) markers. Continental tall fescue accessions are coloured green and Continental tall fescue accessions with an alternate chloroplast DNA *matK* haplotype are dark green. Mediterranean tall fescue accessions are coloured blue and rhizomatous tall fescue accessions, pink. Scale bars represent the dissimilarity index as determined using the Dice coefficient.

In order to assess the utility of the validated SNPs, recorded allele scores for 64 polymorphic loci from the SNaPshot™ assay were used to estimate the genetic similarities of the tall fescue genotypes within the validation panel (Figure 2). The resulting phenogram was largely congruent with an UPGMA phenogram constructed using allele data from SSR markers which were designed from perennial ryegrass DNA template. Both phenograms were able to define the three morphotypes of tall fescue which are represented by the three largest clusters. However in both cases, single Mediterranean (PI 232879) and rhizomatous (PI 423044) genotypes clustered with the Continental tall fescue genotypes rather than those of their own morphotype grouping. Four genotypes representing a genetic sub-group of Continental tall fescue (as defined by an alternate *matK* sequence haplotype), were included in the validation panel, and three of these genotypes were separated from the remaining Continental tall fescue genotypes within the SNP-based phenogram. Higher resolution of the genetic relationships was achieved through the SSR alleles, which generated larger dissimilarity coefficients, as was most evident within the Continental morphotype cluster. Nevertheless, SNPs validated in the present study were able to be confidently used to study genetic relationships within tall fescue. An increase in the number of SNPs used would be anticipated to reduce the disparity in resolution between the SNP- and SSR-based phenograms.

### Attribution of SNPs to tall fescue sub-genomes

**Figure 3:**
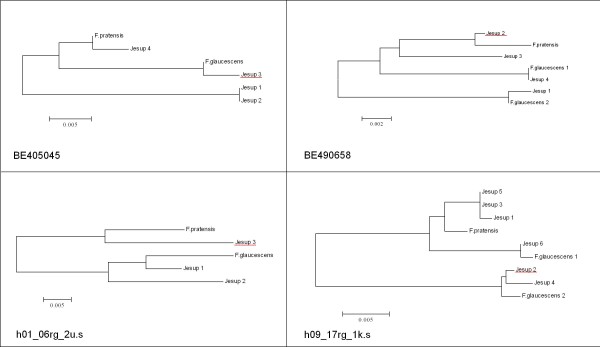
**Attribution of SNPs to sub-genomes.** Examples of neighbour joining trees that were constructed to identify the sub-genome origin of each validated SNP. The consensus sequence of putative sub-genomes from Jesup were aligned with the orthologous haplotypes from the progenitor species meadow fescue (*F. pratensis –* P sub-genome) and tetraploid tall fescue (*F. glaucescens* – G sub-genomes). The haplotype in which the SNP was identified is underlined in red. In these examples, the SNP was attributed to the P sub-genome (SNPs BE490658, h01_06rg_2u.s) and one of the G sub-genomes (SNPs BE405045, h09_17rg_1k.s).

As a proof of concept, attribution of validated SNPs to a sub-genome within hexaploid tall fescue was undertaken by assessing the phylogeny of each putative sub-genome consensus sequence with its orthologous counterpart from meadow fescue and *F. arundinacea* var. *glaucescens*. This approach was implemented for 80 amplicons, each containing a SNP validated within the Continental tall fescue morphotype. For each SNP-containing amplicon, contigs were formed using each putative sub-genome consensus sequence from cv. Jesup, along with sequence from the two progenitor species, meadow fescue and *Festuca arundinacea* var. *glaucescens*. Neighbour joining trees could not be constructed for 9 amplicons, as putative sub-genome consensus sequences within these contigs failed to overlap. Of the remaining amplicons, the majority of contigs formed (38) contained meadow fescue sequence and a single *F. arundinacea* var. *glaucescens* haplotype. Contigs for only two amplicons contained more than one contribution from the tetraploid progenitor, while 19 contigs contained only a representative sequence from the meadow fescue progenitor and were therefore less informative. Based on relationships within the neighbour joining trees, 27 SNPs were attributed to the P sub-genome (most closely related to contemporary meadow fescue) and 13 SNPs to either of the two resident G (G_1_, G_2_) sub-genomes (most closely related to contemporary *F. arundinacea* var. *glaucescens*). A clear affinity to either the P or either G sub-genome could not be achieved for 10 SNP loci, which may represent polymorphisms within the second G sub-genome not represented within the contig. Examples of neighbour joining trees used for sub-genome attribution are shown in Figure 3.

## Discussion

In order to improve rates of genetic gain in plant breeding, a large number of SNPs are required for GWAS or GS approaches in crop species, particularly for those with a limited extent of linkage disequilibrium [37]. A further requirement for such studies is that SNP loci are regularly distributed throughout the whole genome. For allopolyploid species, this requirement extends to the presence of SNPs within each resident sub-genome, in addition to locations along each chromosome. The objective of the present study was to develop and implement a novel bioinformatic pipeline to enable identification of SNPs within amplicons distributed throughout the allohexaploid tall fescue genome, and more specifically, to predict and validate the SNPs within each of the three sub-genomes.

### Comparative genomics for genome-wide SNP locus coverage

For tall fescue, which remains a poorly characterised species with respect to available genomic resources, the targeting of genome-wide distributed regions for resequencing is best achieved using a comparative genomics approach. The methods used here, employing wheat and perennial ryegrass ESTs and the complete *B. distachyon* genome sequence, rely upon the known macrosynteny between the Triticeae cereals, Poeae grasses (including perennial ryegrass) and *B. distachyon*[29,38-40]. While hexaploid tall fescue itself has not been the subject of any studies of synteny within the grasses, it can be assumed with a reasonable level of confidence, that the blocks of synteny observed in this study will also be conserved within tall fescue. Conserved genome co-linearity has been reported, at the genetic map level, between perennial ryegrass and meadow fescue [40] and is therefore likely to also exist between perennial ryegrass and hexaploid tall fescue, given that meadow fescue is a diploid progenitor species of at least the Continental morphotype of tall fescue [24,25]. Less certainty exists, however, for the conservation of these synteny blocks within Mediterranean hexaploid tall fescue, given its less characterised genomic constitution [25]. Although the precise location of the sequenced regions within the hexaploid tall fescue genome (of either morphotype) may only be determined through genetic or physical mapping in the future, the prior comparative genomics analysis has at least increased the probability that the discovered SNPs are distributed throughout the genome.

### Prediction of sub-genome specific SNPs

The SNP discovery approach taken in this study exploited the higher prevalence of HSVs as compared to SNPs within the tall fescue genome, and by hence assembling sequence reads at an elevated stringency, has generated contigs representative of putative individual sub-genomes. This process aimed to reduce the haplotype complexity within contigs to a level comparable to SNP discovery within an outbreeding diploid species [41]. For hexaploid tall fescue, presence of HSVs would ideally allow each amplicon to be resolved into three contigs representing each sub-genome. However, in practice, from one to as many as nine contigs per amplicon were observed. Observation of fewer than three contigs per amplicon probably indicates either an insufficient number of HSVs present within the sequenced region to decompose the contigs into individual haplotypes, or substantial divergence between sub-genomes in the primer binding sites, such that not all sub-genomes were initially amplified. The presence of greater than three haplotypes is compatible with a number of explanations, the first being that multiple paralogous gene copies were the targets of amplification and sequencing. This hypothesis is supported by sequencing data from the diploid meadow fescue, for which 64 amplicons displayed evidence of the presence of paralogues. Another possible explanation is that sequencing error was responsible for introducing sequence variants capable of generating additional contigs, particularly given the reputation of 454 sequencing for introducing homopolymer indel-like errors [42]. Also, if a sufficient number of allelic SNPs were present within a particular sequenced region, this variation may have been responsible for generating more than three contigs per amplicon. Finally, as the amplicon set was sheared prior to sequencing, multiple contigs per amplicon were also formed as a result of gaps within the alignment, for which 454 reads failed to cover the length of the amplicon. This occurrence would be reduced if sequencing depth was increased per amplicon. As a consequence of all these factors, it is unlikely that the implemented approach has succeeded in generating contigs consistently representing only each resident sub-genome. However, alignment at an elevated stringency achieved useful reduction of haplotype complexity and increased the probability of identifying genuine polymorphic SNPs [19].

The number of studies detailing SNP discovery for other allopolyploid species using next-generation sequencing data is increasing, but is still currently limited. The process of SNP-HSV discrimination [19] is simplified in homozygous polyploid species such as self-pollinating wheat [43] and oat [13] and species capable of generation of double haploid lines, including canola [44]. In these instances, any intra-genomic variation can be assumed to represent the presence of HSVs. Bundock et al. (2009) [14] used minor allele frequencies (MAFs) to select probable allelic SNPs when 454 technology was used to sequence amplicons from sugarcane, a highly heterozygous heteropolyploid species. While the use of MAFs for SNP prediction is also possible for tall fescue, the approach ultimately used here has the advantage of generating sub-genome-specific template sequences which can be used for the design of highly multiplexed SNP assays such as Illumina GoldenGate™. A similar approach to that described here, based upon the reduction of haplotype complexity through the separate assembly of homoeologs, has recently been reported in allotetraploid cotton [45]. In this instance, a sub-genome of origin was able to be attributed to many SNPs and consequently, upon linkage mapping, to the cotton homoeologous groups, demonstrating the success of this SNP discovery approach.

It was anticipated that a large collection of SNPs would be identified between the tall fescue genotypes used in this study, given this is a highly heterozygous species, a consequence of an obligate outbreeding reproductive habit. Furthermore, use of multiple genotypes for the SNP discovery process is likely to yield a larger number than if only two genotypes were used, as is commonly the case when parents of mapping populations are used as template in SNP discovery studies [10,14,44]. Through inclusion of both cultivar and non-domesticated material in the discovery panel for both tall fescue morphotypes, it was anticipated that predicted SNPs will have an enhanced likelihood of utility in breeding applications. This is an important consideration, as inclusion of the target germplasm in the discovery panel will probably ensure that derived SNPs will be transferable, polymorphic and informative in subsequent applications.

### SNP validation

The strategy of reducing haplotype complexity prior to SNP discovery was successful for the identification of polymorphic SNPs, as initially revealed by validation using SNaPshot™ chemistry and subsequently with the GoldenGate™ assay. The predicted SNPs were first tested for polymorphism across a diverse panel of tall fescue genotypes to determine whether conserved HSVs were inadvertently selected. As HSVs represent variation within the tall fescue genome that originates from initial polyploidization events, such variation is likely to be conserved across tall fescue germplasm. Failure to successfully decompose haplotypes within other polyploid species has led to false prediction of SNPs and low validation rates [19,46]. The proportion of polymorphic SNPs identified here (70%, as an average across both validation experiments), however, suggests that genuine allelic variants have indeed been identified. This rate of validation is comparable to that observed in diploid species, which ranges from 59 to 97% in selected studies [12,16,41,47,48]. An interesting result of the validation exercise is the extent of cross-transferability of the SNPs between tall fescue morphotypes. The results from both validation experiments suggest that SNPs may be transferable between morphotypes, however with a success rate of less than 50%. Because of the probable taxonomic distinction between the Continental and Mediterranean morphotypes, common SNPs between the two groups may be more properly regarded as orthologous sequence variants which happen to exhibit, in these instances, complementary allelic variation. Such inter-specific SNP transferability is a relatively under-studied topic in plants, although the current results are consistent with SNP transfer rates observed within the *Eucalyptus* genus for which a decrease in validation rate from c. 80% to 50% was observed when SNPs were assayed from species derived of a different sub-genus [49]. Although a limited level of cross-transfer was observed between tall fescue morphotypes, the result justified the strategy of generating sequence from both morphotypes, rather than relying on the successful inter-morphotype transfer.

Nucleotide variants that were monomorphic within, but polymorphic between morphotypes presumably also correspond to a sub-class of orthologous sequence variants. Although, the identification of such diagnostic features revealed limited success, as during validation, only 4 of 34 tested (12%) proved to be diagnostic within the validation panel. The approach used here probably underestimated the diversity within each morphotype by using the consensus of each putative sub-genome contig for SNP prediction. The number and frequency of SNPs within each tall fescue morphotype is therefore likely to be larger than was originally predicted. The validation process did, however, inadvertently identify a further 5 nucleotide variants capable of differentiation between either the Continental and Mediterranean or Continental and rhizomatous morphotypes, demonstrating the need to evaluate assays across diverse germplasm before implementation as either a polymorphic or diagnostic marker.

Alternatively, haplotypes based on multiple SNP alleles are likely to be an effective means for discrimination of morphotypes, as shown by the SNP-based phenogram, which was able to provide resolution comparable with the phenogram constructed using SSR-derived data. The level of concordance between the two phenograms also demonstrates the utility of these SNPs. Although both depict similar genetic relationships within the validation panel, the 302 SSR alleles provided greater resolution, particularly within the Continental accessions. As SNPs are generally only biallelic in nature, it has been estimated that c. 1,000 SNP loci are required to achieve the same level of resolution in diversity studies as that attained by c. 100 SSRs [50]. For the purpose of SNP-based diagnostics in tall fescue, therefore, it may be more reliable to use nucleotide variants identified previously in conserved regions of the chloroplast DNA-derived gene *matK* and the internal transcribed spacer region of ribosomal DNA, as implemented in a previous study [26].

### Quantitative genotyping

This study has also demonstrated the quantitative genotyping capabilities of the GoldenGate™ assay for an outbreeding hexaploid species. The potential of this assay for allohexaploid species has been previously explored in wheat [51], but as homozygous individuals were used, only two genotype clusters for a locus-specific assay were ever expected in that particular study. The results presented here represent the first time, to our knowledge, that the GoldenGate™ assay has successfully been used for co-dominant genotyping of SNP loci in an outbreeding hexaploid. These results demonstrate that SNP genotyping is most effective in those instances in which a polymorphism is present in a single sub-genome and no polymorphism is present at the corresponding loci on the homoeologous chromosomes. In these instances, three possible genotypes may arise (i.e. AAAAAA, ABAAAA and BBAAAA) and from some SNP loci, the assay has proven sensitive enough to distinguish each class based on nucleotide variant dosage. An even less complicated scenario has also been achieved in which only the SNP-containing sub-genome appears to be amplified, such that the genotyping assay effectively scores a diploid constitution (i.e. AA, AB, BB). It seems probable that this scenario was made possible by provision of putative sub-genome specific templates to Illumina for primer design. This equivalent approach of complexity reduction by amplification from a single sub-genome has been reported on a smaller scale in bread wheat [52,53]. However, the combination of the bioinformatic SNP discovery process used here, and the Illumina GoldenGate™ assay has demonstrated that this outcome can also be achieved in a high-throughput manner.

Differences between the two genotyping chemistries utilised in this work were demonstrated by the comparison of allele calls for 51 different SNP loci. As each chemistry utilises different primer sequences for SNP locus amplification, it is understandable that some degree of discordance would be observed. For example, SNPs observed as monomorphic within the GoldenGate™ assay were frequently classed as polymorphic when assayed using SNaPshot chemistry. In this instance, it is possible that the primer sequences designed by Illumina failed to target the relevant sequence variant, which may be a disadvantageous consequence of supplying putative sub-genome specific sequence templates for primer design. The clarity of the clusters formed in the GoldenGate™ assay also influenced the concordance of allele calls and hence the reliability of the SNP. Clustering was more diffuse for some SNPs which may reflect a more complicated amplification scenario, in which dosage-based allele discrimination was less successful. In these instances, allele calling was not as reliable, resulting in the lower level of concordance between the two genotyping chemistries. In summary, based on this comparison, the most reliable and useful SNPs appear to be those which either form three distinct clusters or are diagnostic for specific morphotypes.

It also appears in some instances, that the same nucleotide polymorphism may be present at the same homeoloci across multiple sub-genomes, a scenario leading to more than three clear genotype calls. Despite this complexity, data analysis permitted full genotypic resolution, as was the case for two SNP loci, when five clusters were identified, corresponding to polymorphism in either or both of two sub-genomes (i.e. AAAAAA, AAABAA, AABBAA, ABBBAA, BBBBAA). Alternatively, the generation of this number of clusters may be due to primer binding to a polymorphic region, causing non-uniform clustering of the included genotypes.

### Attribution of SNPs to sub-genomes

An advantageous consequence of identifying SNPs within individual sub-genomes of an allopolyploid, is the ability to then attribute each locus to the resident sub-genome of origin. In other polyploid species, this outcome has been achieved either through the use of characterised aneuploid (such as nullisomic-tetrasomic substitution) lines (wheat) [18,54] or by comparison of the generated sequence with that obtained from the diploid progenitor species, if the identities of these taxa are known (as for white clover and cotton) [45,55,56]. Unlike for wheat, substitution lines are not yet readily available for tall fescue. Consequently, assignment of sequence haplotypes to individual sub-genomes can be attempted based on sequence from diploid progenitors, but only to a limited extent. As a hexaploid species, the identity of at least two diploid progenitor species would be required for complete haplotype assignment in tall fescue. Contemporary meadow fescue can fulfil the role as one diploid progenitor, but the two diploid species that hybridised to generate modern day allotetraploid *F. arundinacea* var. *glaucescens* have not as yet been identified [25]. Phylogenetic inferences in this study have therefore been able to only assign validated SNPs with confidence to the P (meadow fescue-related) sub-genome. Nevertheless, the results have indicated a clear bias of validated SNPs towards origin from the P genome compared to the G_1_ or G_2_ sub-genomes. This result may be a consequence of preferential amplification from this sub-genome when the amplicon libraries were made, given that the majority of primers used were designed from perennial ryegrass genome template sequence, which is likely to have a higher identity to the P sub-genome than either the G_1_ or G_2_ sub-genomes [25]. Alternatively, the larger proportion of P sub-genome-specific SNPs may represent a genuine biological phenomenon of higher polymorphism within this sub-genome.

Although sequences flanking a number of SNP loci clearly showed a higher affinity to the *F. arundinacea* var. *glaucescens* derived sequence, the identification of each haplotype as derived from either the G_1_ or G_2_ sub-genomes cannot consistently be confirmed. For the validated SNPs examined here, only 2 contigs contained multiple *F. arundinacea* var. *glaucescens* haplotypes, assumed to be representative of both sub-genomes. The deficit of multiple haplotypes derived from the G sub-genomes is possibly a result of obtaining an insufficient number of 454 reads for the *F. arundinacea* var. *glaucescens* amplicons, as previous work has demonstrated that sufficient sequence variation exists between the G_1_ and G_2_ sub-genomes for differentiation [25]. Given these current limitations in the identification of sub-genome-specific haplotypes, validated SNPs could instead be assigned to locations on a tall fescue genetic linkage map to enhance understanding of their sub-genome of origin. For this purpose, future work could involve the development of a mapping population, generated using two of the genotypes within the SNP discovery panel as parents.

### Future applications

The collection of SNPs developed in this work is likely to prove useful for applications such as cultivar identification and discrimination, genetic linkage map construction and could also be implemented as part of any future GWAS and GS based molecular breeding approaches in tall fescue. However, while this work represents a substantial advancement in SNP development for tall fescue, a much higher marker density is anticipated to be required for both GWAS and GS applications. Given the obligate outbreeding habit of this species, the predicted degree of linkage disequilibrium is likely to require a higher marker density for GWAS or GS in tall fescue compared to inbreeding species such as barley or rice [5,57]. In order to generate a larger number of SNP markers at a higher density throughout the tall fescue genome, alternative high-throughput SNP discovery approaches such as genotyping by sequencing (GBS) could be performed. Unlike amplicon sequencing, the various current methods for GBS are not restricted by a need for prior sequence knowledge to support primer design, and can potentially generate hundreds of thousands of marker loci [58,59]. It is likely that such alternative SNP discovery approaches will be necessary to ultimately permit GWAS and GS breeding strategies in tall fescue. However, regardless of the methodology used for SNP discovery in tall fescue, the haplotype complexities created by the allohexaploid genome structure will still need to be addressed, and the approach taken in this study is applicable to data from multiple sequencing methodologies.

## Conclusions

This study has described the development of a bioinformatic pipeline which was used to generate a collection of SNP markers that are likely to be distributed throughout the genome of tall fescue. SNP resources were developed for both of the major tall fescue morphotypes (Continental and Mediterranean). Based on validation data, it can be predicted that of the 8,584 and 2,292 high-confidence SNPs identified for the Continental and Mediterranean morphotypes respectively, approximately two-thirds will be polymorphic and informative, while approximately one-third will be capable of co-dominant scoring using the GoldenGate™ assay. In order to implement such SNPs for tall fescue molecular breeding, the next immediate action would be to refine the collection such that only polymorphic, co-dominant scoreable SNPs are present in a customised GoldenGate™ panel. This finalised SNP collection could then be used to genotype tall fescue individuals in effectively the same manner as would be performed for a diploid species, mitigating the difficulties associated with SNP genotyping in an allohexaploid system. Such a collection of SNPs distributed throughout the genome would be useful for numerous applications, including cultivar identification and discrimination, genetic linkage map construction, and would provide at least a starting point for the implementation of GWAS and GS in breeding of each tall fescue morphotype.

## Competing interests

The authors declare that they have no competing interests.

## Authors’ contributions

MH performed the sample preparation, 454 sequencing, data assembly, SNP identification, SNP validation and drafted the manuscript. NC and JF co-conceptualised and coordinated the project and assisted in drafting the manuscript. All authors read and approved the final manuscript.

## Supplementary Material

Additional file 1 **SNP validation panels.** Details of the tall fescue genotypes used for SNP validation with SNaPshot™ and GoldenGate™ chemistries. Accessions described as ‘Continental 2’ represent those of the Continental morphotype that have been shown to possess alternate *matK* sequence haplotype. Click here for file

Additional file 2 **List of wheat ESTs.** Unique identifiers of the 674 wheat deletion bin-mapped ESTs used for comparative genomics. Sequence from all the ESTs can be accessed from GenBank. Click here for file

Additional file 3 **Graphical display of conserved genomic regions between wheat and *B. distachyon.*** Individual green diamonds represent a deletion bin-mapped wheat EST, and its position upon the x-axis indicates the location of its orthologue within *B. distachyon*, as determined by BLASTN analysis. The x-axis represents the 5 chromosomes of *B. distachyon* and the y-axis represents the 7 wheat chromosomes. The location of each wheat EST within the wheat chromosome is resolved to the level of chromosome arm, with the short (S) and long (L) arms separated on the y-axis. The blocks of synteny targeted for primer design are listed in Additional file 4. Click here for file

Additional file 4 **Details of 19 predicted synteny blocks identified between the*B. distachyon*and wheat genomes.** A list of the 19 predicted synteny blocks detailing their size, the number of perennial ryegrass ESTs thought to be located within each block (entries), and the number of perennial ryegrass ESTs selected for primer design within each block. Chr = chromosome, Bd = *Brachypodium distachyon*. Click here for file

Additional file 5 **Predicted distribution of amplicons selected for resequencing.** The predicted chromosomal locations of the amplicons selected for resequencing, based upon similarity to mapped wheat ESTs. Click here for file

Additional file 6 **SNPs used for validation.** Details of the SNPs used for validation with SNaPshot™ and GoldenGate™ chemistries including the locus-specific amplification primers, interrogation primers and flanking sequences. Accessions described as ‘Continental 2’ are as described in the legend to Additional file 1. Click here for file

Additional file 7 **Allele designations generated using SNaPshot™ chemistry.** Allele calls generated for each tall fescue genotype included in the SNaPshot™ SNP validation assay. Accessions described as ‘Continental 2’ are as described in the legend to Additional file 1. Click here for file

Additional file 8 **Allele designations generated using GoldenGate™ chemistry.** Allele calls generated for each tall fescue genotype included in the GoldenGate™ SNP validation assay. Accessions described as ‘Continental 2’ are as described in the legend to Additional file 1. Click here for file
