# Supplementary material for: Genome-wide SNP identification in multiple morphotypes of allohexaploid tall fescue (Festuca arundinacea Schreb)
Source: BMC Genomics. 2012 Jun 6;13:219. doi: 10.1186/1471-2164-13-219 (PMC3444928; doi:10.1186/1471-2164-13-219)
Supplement: Additional file 1 — SNP validation panels. Details of the tall fescue genotypes used for SNP validation with SNaPshot™ and GoldenGate™ chemistries. Accessions described as ‘Continental 2’ represent those of the Continental morphotype that have been shown to possess alternate matK sequence haplotype. [file 1471-2164-13-219-S1.doc]

| **Accession/cultivar** | **Morphotype** | **Validation using SNaPshot^TM^ assay** | **Validation using GoldenGate^TM^ assay** |
| --- | --- | --- | --- |
| 143045 | Continental |  | ✓ |
| 227446 | Continental | ✓ | ✓ |
| 234884 | Continental | ✓ | ✓ |
| 234890 | Continental |  | ✓ |
| 277839 | Continental | ✓ | ✓ |
| 277840 | Continental | ✓ | ✓ |
| 277843 | Continental |  | ✓ |
| 283293 | Continental | ✓ | ✓ |
| 283296 | Continental |  | ✓ |
| 283301 | Continental |  | ✓ |
| 287820 | Continental | ✓ | ✓ |
| 304844 | Continental | ✓ | ✓ |
| 323256 | Continental |  | ✓ |
| 418599 | Continental |  | ✓ |
| 418603 | Continental | ✓ | ✓ |
| 418696 | Continental |  | ✓ |
| 422628 | Continental | ✓ | ✓ |
| 422633 | Continental |  | ✓ |
| 422655 | Continental |  | ✓ |
| 422663 | Continental | ✓ | ✓ |
| 422714 | Continental | ✓ | ✓ |
| 422717 | Continental |  | ✓ |
| 422732 | Continental | ✓ | ✓ |
| 422743 | Continental | ✓ | ✓ |
| 422752 | Continental |  | ✓ |
| 422764 | Continental |  | ✓ |
| 423052 | Continental | ✓ | ✓ |
| 423054 | Continental |  | ✓ |
| 423069 | Continental |  | ✓ |
| 423087 | Continental | ✓ | ✓ |
| 423093 | Continental |  | ✓ |
| 423108 | Continental |  | ✓ |
| 423116 | Continental | ✓ | ✓ |
| 502368 | Continental |  | ✓ |
| 504538 | Continental | ✓ | ✓ |
| 505832 | Continental | ✓ | ✓ |
| 512309 | Continental |  | ✓ |
| 514633 | Continental |  | ✓ |
| 527494 | Continental | ✓ | ✓ |
| 527495 | Continental | ✓ | ✓ |
| 574522 | Continental |  | ✓ |
| 596701 | Continental | ✓ | ✓ |
| 598929 | Continental |  | ✓ |
| 598944 | Continental | ✓ | ✓ |
| 601279 | Continental |  | ✓ |
| 608025 | Continental |  | ✓ |
| 619026 | Continental | ✓ | ✓ |
| 634234 | Continental | ✓ | ✓ |
| 634236 | Continental |  | ✓ |
| 636591 | Continental | ✓ | ✓ |
| 636598 | Continental |  | ✓ |
| 639782 | Continental |  | ✓ |
| Jesup | Continental | ✓ | ✓ |
| KY31 | Continental | ✓ | ✓ |
| Quantum | Continental | ✓ | ✓ |
| 174209 | Continental2 |  | ✓ |
| 277838 | Continental2 |  | ✓ |
| 311044 | Continental2 |  | ✓ |
| 315433 | Continental2 |  | ✓ |
| 440363 | Continental2 | ✓ | ✓ |
| 499495 | Continental2 |  | ✓ |
| 505833 | Continental2 | ✓ | ✓ |
| 619025 | Continental2 | ✓ | ✓ |
| 636667 | Continental2 | ✓ | ✓ |
| 208681 | Mediterranean |  | ✓ |
| 231553 | Mediterranean | ✓ | ✓ |
| 231555 | Mediterranean |  | ✓ |
| 232879 | Mediterranean | ✓ | ✓ |
| 255875 | Mediterranean |  | ✓ |
| 261029 | Mediterranean | ✓ | ✓ |
| 269850 | Mediterranean | ✓ | ✓ |
| 283280 | Mediterranean |  | ✓ |
| 287819 | Mediterranean | ✓ | ✓ |
| 297907 | Mediterranean |  | ✓ |
| 347572 | Mediterranean |  | ✓ |
| 535582 | Mediterranean | ✓ | ✓ |
| 598924 | Mediterranean |  | ✓ |
| 598932 | Mediterranean | ✓ | ✓ |
| 598934 | Mediterranean |  | ✓ |
| 610943 | Mediterranean | ✓ | ✓ |
| 610952 | Mediterranean | ✓ | ✓ |
| 610956 | Mediterranean |  | ✓ |
| 619480 | Mediterranean | ✓ | ✓ |
| 634229 | Mediterranean |  | ✓ |
| 636536 | Mediterranean |  | ✓ |
| 636537 | Mediterranean |  | ✓ |
| Prosper | Mediterranean | ✓ | ✓ |
| Resolute | Mediterranean | ✓ | ✓ |
| 231563 | Rhizomatous |  | ✓ |
| 287821 | Rhizomatous |  | ✓ |
| 423044 | Rhizomatous | ✓ | ✓ |
| 423129 | Rhizomatous |  | ✓ |
| 512306 | Rhizomatous |  | ✓ |
| 512314 | Rhizomatous |  | ✓ |
| 512315 | Rhizomatous | ✓ | ✓ |
| 578716 | Rhizomatous | ✓ |  |
| Torpedo | Rhizomatous | ✓ | ✓ |
