# Supplementary material for: Genome-wide SNP identification in multiple morphotypes of allohexaploid tall fescue (Festuca arundinacea Schreb)
Source: BMC Genomics. 2012 Jun 6;13:219. doi: 10.1186/1471-2164-13-219 (PMC3444928; doi:10.1186/1471-2164-13-219)
Supplement: Additional file 4 — Details of 19 predicted synteny blocks identified between theB. distachyonand wheat genomes. A list of the 19 predicted synteny blocks detailing their size, the number of perennial ryegrass ESTs thought to be located within each block (entries), and the number of perennial ryegrass ESTs selected for primer design within each block. Chr = chromosome, Bd = Brachypodium distachyon. [file 1471-2164-13-219-S4.doc]

| **Block** | **Bd**  **Chr** | **Wheat**  **HG** | **Wheat HG**  **Arm** | **Entries** | **No. perennial ryegrass**  **ESTs selected** | **Bd Chr**  **Start (bp)** | **Bd Chr**  **End (bp)** | **Block size**  **(Mbp)** |
| --- | --- | --- | --- | --- | --- | --- | --- | --- |
| 1 | 1 | 5 | Long | 1476 | 22 | 1475516 | 12292122 | 10.8 |
| 2 | 1 | 4 | Short | 627 | 9 | 6678199 | 11198610 | 4.5 |
| 3 | 1 | 2 | Short | 1436 | 18 | 13395147 | 22557944 | 9.2 |
| 4 | 1 | 7 | Long | 2280 | 35 | 21504810 | 38654916 | 17.2 |
| 5 | 1 | 7 | Short | 1027 | 26 | 40103633 | 52961643 | 12.9 |
| 6 | 1 | 4 | Long | 2125 | 96 | 59302141 | 74411483 | 15.1 |
| 7 | 2 | 3 | Short | 1076 | 23 | 48798 | 11859919 | 11.8 |
| 8 | 2 | 1 | Long | 1443 | 34 | 10125233 | 27177104 | 17.1 |
| 9 | 2 | 1 | Short | 603 | 15 | 32493283 | 39852607 | 7.4 |
| 10 | 2 | 3 | Long | 1909 | 192 | 49512 | 11803522 | 11.8 |
| 11 | 3 | 6 | Short | 941 | 18 | 343463 | 9511797 | 9.2 |
| 12 | 3 | 7 | Long | 655 | 15 | 11605066 | 19023764 | 7.4 |
| 13 | 3 | 1 | Long | 660 | 28 | 22830216 | 36675308 | 13.8 |
| 14 | 3 | 7 | Short | 589 | 14 | 39284910 | 46563304 | 7.3 |
| 15 | 3 | 6 | Long | 1441 | 24 | 47582544 | 59626369 | 12.0 |
| 16 | 4 | 5 | Short | 485 | 11 | 232913 | 5983215 | 5.8 |
| 17 | 4 | 4 | Short | 184 | 7 | 27826652 | 31241655 | 3.4 |
| 18 | 4 | 5 | Long | 1470 | 36 | 29440830 | 47374364 | 17.9 |
| 19 | 5 | 2 | Long | 1463 | 45 | 6129438 | 28287359 | 22.2 |

HG = homeologous group
