# Supplementary material for: Genome-wide SNP identification in multiple morphotypes of allohexaploid tall fescue (Festuca arundinacea Schreb)
Source: BMC Genomics. 2012 Jun 6;13:219. doi: 10.1186/1471-2164-13-219 (PMC3444928; doi:10.1186/1471-2164-13-219)
Supplement: Additional file 5 — Predicted distribution of amplicons selected for resequencing. The predicted chromosomal locations of the amplicons selected for resequencing, based upon similarity to mapped wheat ESTs. [file 1471-2164-13-219-S5.doc]

|  | **Wheat homoeologous group** | | | | | | |
| --- | --- | --- | --- | --- | --- | --- | --- |
|  | **1** | **2** | **3** | **4** | **5** | **6** | **7** |
| Short arm | 11 | 13 | 16 | 12 | 6 | 13 | 16 |
| Long arm | 36 | 35 | 103 | 82 | 40 | 7 | 24 |
|  | Number of amplicons | | | | | | |
