# Supplementary material for: Genome-wide SNP identification in multiple morphotypes of allohexaploid tall fescue (Festuca arundinacea Schreb)
Source: BMC Genomics. 2012 Jun 6;13:219. doi: 10.1186/1471-2164-13-219 (PMC3444928; doi:10.1186/1471-2164-13-219)
Supplement: Supplementary file 5 [file 1471-2164-13-219-S3.doc]

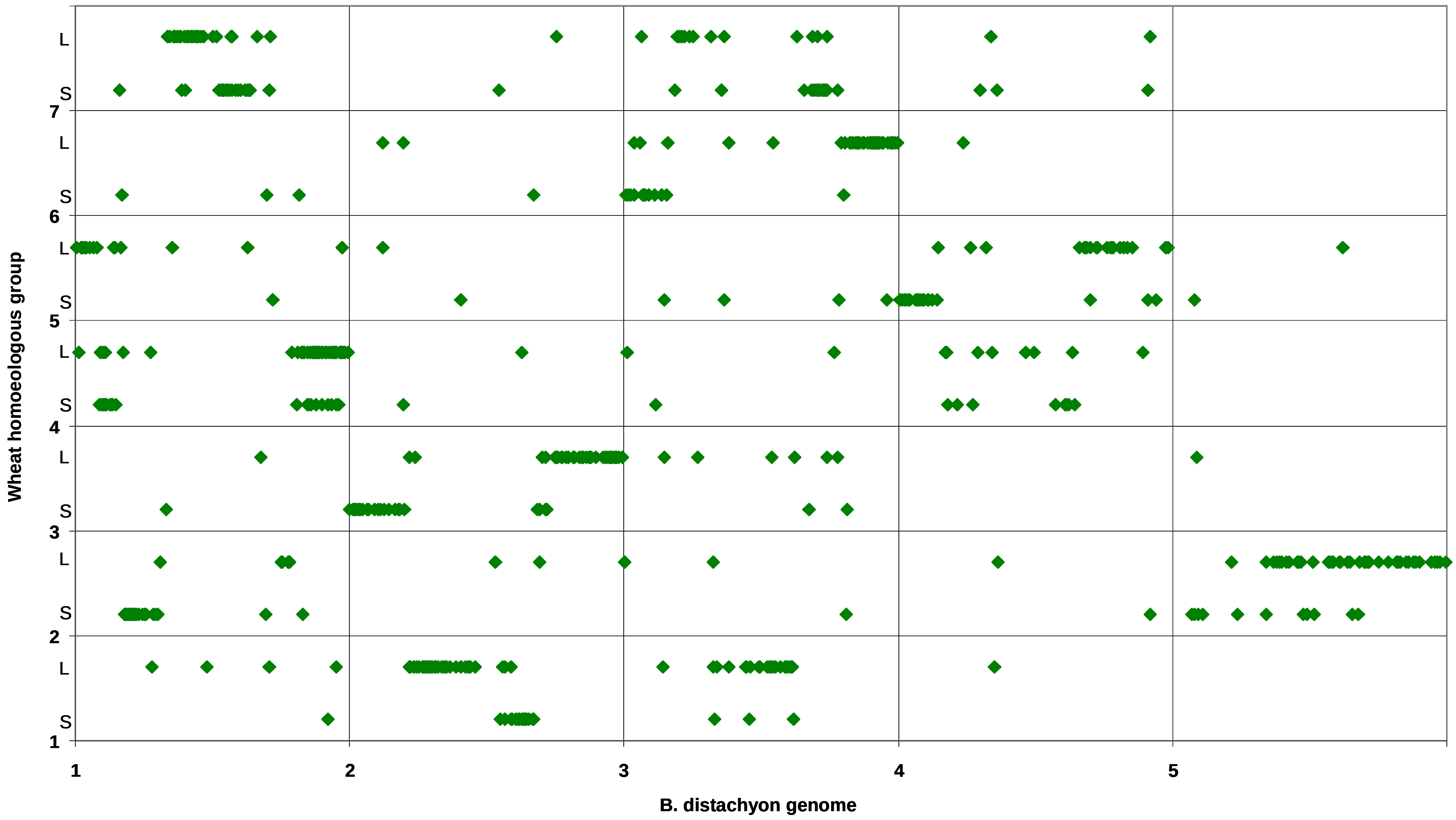

Wheat homoeologous group
L
S
7
L
S
6
L
S
5
L
S
4
L
S
3
L
S
2
L
S
1
1
2
3
4
5
B. distachyon genome
